# Acute airway obstruction due to postoperative retropharyngeal hematoma after anterior cervical fusion: a retrospective analysis

**DOI:** 10.1186/s13018-017-0517-z

**Published:** 2017-01-26

**Authors:** Kyung-Jin Song, Byung-Wan Choi, Dong-Hyun Lee, Dong-Ju Lim, Seung-Yeol Oh, Sung-Soo Kim

**Affiliations:** 1 Department of Orthopaedic Surgery, Chonbuk National University Medical School, Jeonju, Korea; 2 Department of Orthopaedic Surgery, Inje University Haeundae Paik Hospital, Haeundae-ro 875, Haeundae-gu, Busan, 48108 Korea; 3 Seoul Spine Institute, Inje University Sanggye Paik Hospital, Seoul, Korea

**Keywords:** Cervical spine, Anterior servical fusion, Acute airway obstruction, Hematoma

## Abstract

**Background:**

Acute airway obstruction (AAO) after anterior cervical fusion (ACF) can be caused by postoperative retropharyngeal hematoma, which requires urgent recognition and treatment. However, the causes, evaluation, and appropriate treatment of this complication are not clearly defined. The purpose of this retrospective review of a prospective database was to investigate etiologic factors related to the development of AAO due to postoperative hematoma after ACF and formulate appropriate prevention and treatment guidelines.

**Methods:**

Cervical spinal cases treated at our academic institutions from 1998 to 2013 were evaluated. Demographic data, including factors related to hemorrhagic tendency, and operative data were analyzed. Patients who developed a hematoma were compared with those who did not to identify risk factors. Cases complicated by hematoma were reviewed, and times until development of hematoma and surgical evacuation were determined. Degrees of airway compromise and patient behavior were classified and evaluated. Treatment was selected according to the patient’s status.

**Results:**

Among 785 ACF procedures performed, there were nine cases (1.15%) of AAO. None of these nine patients had preoperative risk factors. In six patients (67%), the hematoma occurred within 24 h, whereas three patients (33%) presented with hematoma at a median of 72 h postoperatively. Four of the nine patients with AAO underwent evacuation of the hematoma. Two patients with inspiratory stridor, anterior neck swelling, and facial edema progressed to respiratory distress and their hematomas were removed by surgery, during which, sustained superficial venous bleeding was confirmed. Intubation was attempted several times in one patient with cyanosis, but is unsuccessful; cricothyroidotomy was performed in this patient and pumping in the small muscular arterial branches was confirmed in the operating room. All of the patients recovered without any complications.

**Conclusions:**

With rapid recognition and appropriate treatment, there were no long-term complications caused by postoperative hematoma. There were no specific preoperative risk factors for hematoma. Systematic evaluation and appropriate management can be helpful for preventing serious complications after development of a postoperative hematoma.

## Background

Anterior cervical decompression and fusion (ACF) is a generally performed surgical procedure for symptomatic radiculopathy of the cervical spine and myelopathy. Since its initial description and application by Robinson and Smith [1], long-term data indicate that this technique produces excellent results and it is now used extensively. Various ACF-related complications have been reported, including neurologic injury, vascular injury, postoperative infection, mechanical injury, and instrument-related problems [2–5].

Acute airway obstruction (AAO) caused by a hematoma that compromises an airway is the most serious and fatal complication of ACF. The reported incidence of this complication ranges from 0.2 to 1.9% [5, 6]. Currently, no report has clearly elucidated the etiology, evaluation, treatment, or means of preventing AAO caused by a hematoma after ACF. Appropriate evaluation and treatment guidelines are required to prevent the airway complications of ACF. The objective of this study was to investigate the incidence of postoperative hematoma after ACF and the risk factors for its occurrence. With a better understanding of the risk factors involved, potential strategies to avoid this life-threatening complication could be formulated. This study also attempted to develop a systematic evaluation process, an appropriate therapeutic approach, and ways to prevent the complications of AAO.

## Methods

Patients with acute respiratory failure were identified from a total of 785 patients who had undergone ACF and had at least 1 year of follow-up at our institutions from October 1998 to June 2013. Patients with posterior fusion or anterior-posterior fusion were excluded. Nine patients (seven males, two females) developed AAO subsequent to a postoperative hematoma. The median patient age was 62 years. Among the nine cases, there were five cases of traumatic injury and four of degenerative disease. We analyzed a number of preoperative and intraoperative factors to evaluate the relationship between ACF and development of postoperative hematoma. The preoperative factors included whether the patient had a bleeding disorder, abnormal hemostasis, a history of using anticoagulants, or liver dysfunction. And also, we checked the blood sample tests like partial thromboplastin time, activated partial thromboplastin time, bleeding time, and coagulation time. Intraoperative factors, such as the amount of bleeding and major vessel damage, were identified in the operation notes. Patients who developed a hematoma were compared with those who did not to identify risk factors.

For the systematic evaluation, we classified AAO according to change in respiratory status, condition of the surgical site, and patient behavior. AAO was classified into three steps according to respiratory status: no severe difficulties in stable breathing, no increase in respiratory rate, and no changes in blood pressure (step 1); an increased respiratory rate, sweating, and respiratory stridor because of aggravating airway compression on inspiration (step 2); and difficulty maintaining breathing accompanied by cyanosis (step 3). The condition of the surgical site was similarly classified into three steps: acute bleeding at the surgical site (step 1), firm edema and smoothing of wrinkles in the neck region because of the aggravating hematoma (step 2), and compromised blood flow to the head with facial edema because of aggravating edema of the neck (step 3). Patients’ reactions were graded as follows: initial agitation because of dyspnea but stabilized with control (grade 1), anxiety and fear that were not easily stabilized because of the aggravating dyspnea (grade 2), and overall reactions decreased because of failure to maintain breathing (grade 3).

To be able to propose a systematic guideline for treatment, we evaluated the treatments provided, i.e., observation, removal of the hematoma, intubation, cricothyroidotomy, and emergency surgery according to the severity of AAO indicated by the steps above.

The statistical analysis was performed using SPSS version 18.0 software (SPSS Inc., Chicago, IL, USA). The Mann-Whitneay test was used to evaluate differences with regard to formation of hematoma. A *p* value <0.05 was deemed to be statistically significant.

## Results

The incidence of AAO was 1.15% (9/785). There were no fatalities or severe complications. None of the patients who developed AAO had preoperative risk factors, such as a bleeding tendency, a hemostatic abnormality, or anticoagulant therapy.

The median amount of blood loss during surgery was 200 (range 150–240) mL, and the median amount of fluid drained after surgery was 80 (range 0–130) mL. On median value, the drain was removed 1 (range 1–3) day after surgery; there was no statistically significant difference in timing of drain removal between the hematoma group and the non-hematoma group (*p* > 0.05).

Classification of respiratory status showed that there were six patients in step 1, two patients in step 2, and one patient in step 3. Evaluation of the surgical site determined that there were three patients in step 1 and four patients in step 2; there were two patients in step 3 and both had facial edema. With regard to patient behavior, there were six patients in step 2 and three patients in step 3.

Five patients who did not undergo surgery improved on supplemental oxygen whilst placed in a sitting position under close observation. Four of the nine patients with dyspnea underwent surgery to remove the hematoma, and their dyspnea was relieved after surgery. In one patient, incorrect insertion of the drain after surgery caused AAO because the hematoma was not appropriately drained and compressed the airway. The patient recovered after removal of the hematoma and reinsertion of the drain. Two patients with inspiratory stridor and facial and neck edema required surgery under general anesthesia to remove the hematoma. One patient had a superficial jugular vein injury and another had an injury to the superior thyroid artery. The hematoma was removed, and the bleeding was controlled in both these patients.

Intubation was attempted to secure one patient’s airway due to cyanosis and a low respiratory rate. However, attempts to intubate the patient failed twice because of laryngeal edema. Therefore, cricothyrotomy was performed immediately and the patient’s breathing improved. After cricothyrotomy, the hematoma was removed in the operating room, and a diffuse intramuscular bleeding thought to be caused by an excessive retraction on the inner muscular layer during the previous operation was cauterized (Table 1). All of the patients recovered without any complications.

**Table 1:** Demographic and clinical details for patients who developed acute airway obstruction after anterior cervical fusion

Age	Sex	Diagnosis	Operation	Medical history	Coagulopathy	Time to onset (Hr)	Respiration status	Excited or panic	Hematoma removal	Cause
63	M	Traumatic HCD	C3-4, 6-7	DM	No	70	Difficult	No	No	
60	M	Traumatic HCD	C3-4	No	No	6	Difficult	No	No	
69	F	Degenerative HCD	C5-7	HTN	No	4	Difficult	Excited	Yes	Dysfunction of drainage
51	M	Traumatic HCD	C4-6	No	No	6	Difficult	No	No	
62	F	Degenerative HCD	C5-6	No	No	8	Difficult	No	No	
67	M	Incomplete cord injury	C5-7	No	No	72	Respiratory stridor	Excited	Yes	Bleeding of the jugular vein
47	M	Degenerative HCD	C5-T1	No	No	8	Cyanosis	Decreased response	Hematoma removal and cricothyroidotomy	Bleeding of the intramuscular vessel
63	M	Degenerative HCD	C3-4	DM	No	4	Respiratory stridor	Panic	Yes	Bleeding of the superior thyroid artery
52	M	Traumatic HCD	C6-7	No	No	120	Difficult	No	No	

*M* male, *F* female, *HCD* herniated cervical disc, *DM* diabetes mellitus, *HTN* hypertension

### Case presentations

#### Case 1

**Fig. 1:**
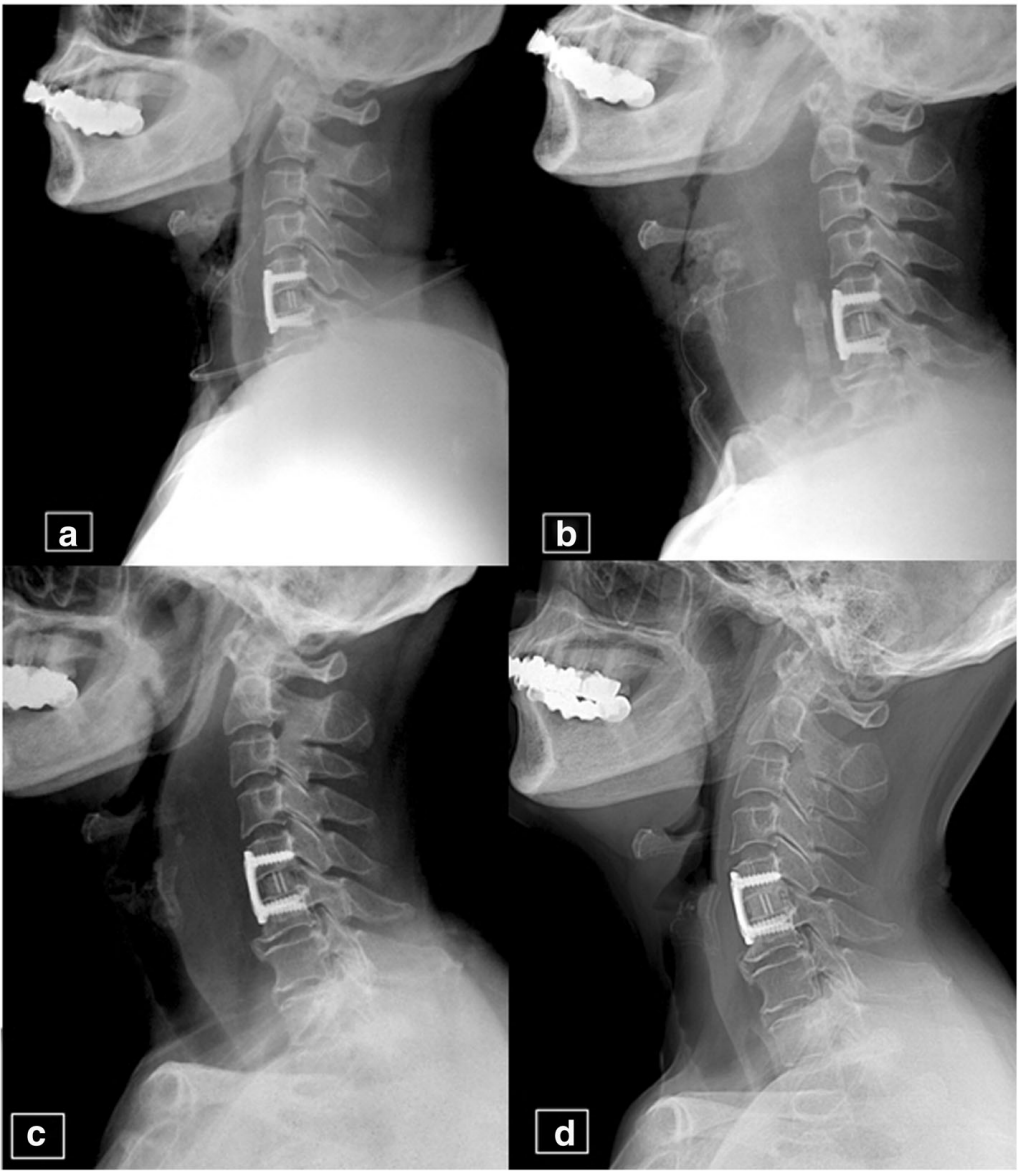
A 67-year-old female patient. **a** Lateral plain radiograph of the cervical spine on the day of operation showing anterior cervical discectomy and fusion at C5-6. **b** Lateral radiograph 12 h postoperatively during acute respiratory failure showing severe prevertebral swelling. **c** Lateral radiograph at 10 days postoperatively showing decreased prevertebral swelling. The patient was discharged without complications. **d** Lateral radiograph at the 12-month follow-up showing no prevertebral swelling

A 67-year-old woman who had had C5-6 anterior cervical discectomy and fusion because of a herniated intervertebral disc developed AAO 12 h after surgery. There were no damaged vessels and an appropriate hemostatic method had been applied during the operation. An X-ray taken when the patient was dyspneic revealed edema in the anterior part of the neck. The diameter of the neck was 3.5 times greater than that seen on an X-ray taken immediately after surgery (Fig. 1a, b). Cyanosis was not identified. Blood pressure was 130/80 mmHg, and the respiratory rate was 22 breaths per minute. The patient was not agitated or anxious but had dyspnea. Although firm edema was found at the surgical site, we decided to observe the patient, provide oxygen, and place her in the sitting position. We prepared the operating room and the surgical team in case her condition deteriorated. The patient’s dyspnea and general condition improved with observation. She was discharged 10 days after surgery (Fig. 1c, d).

#### Case 2

**Fig. 2:**
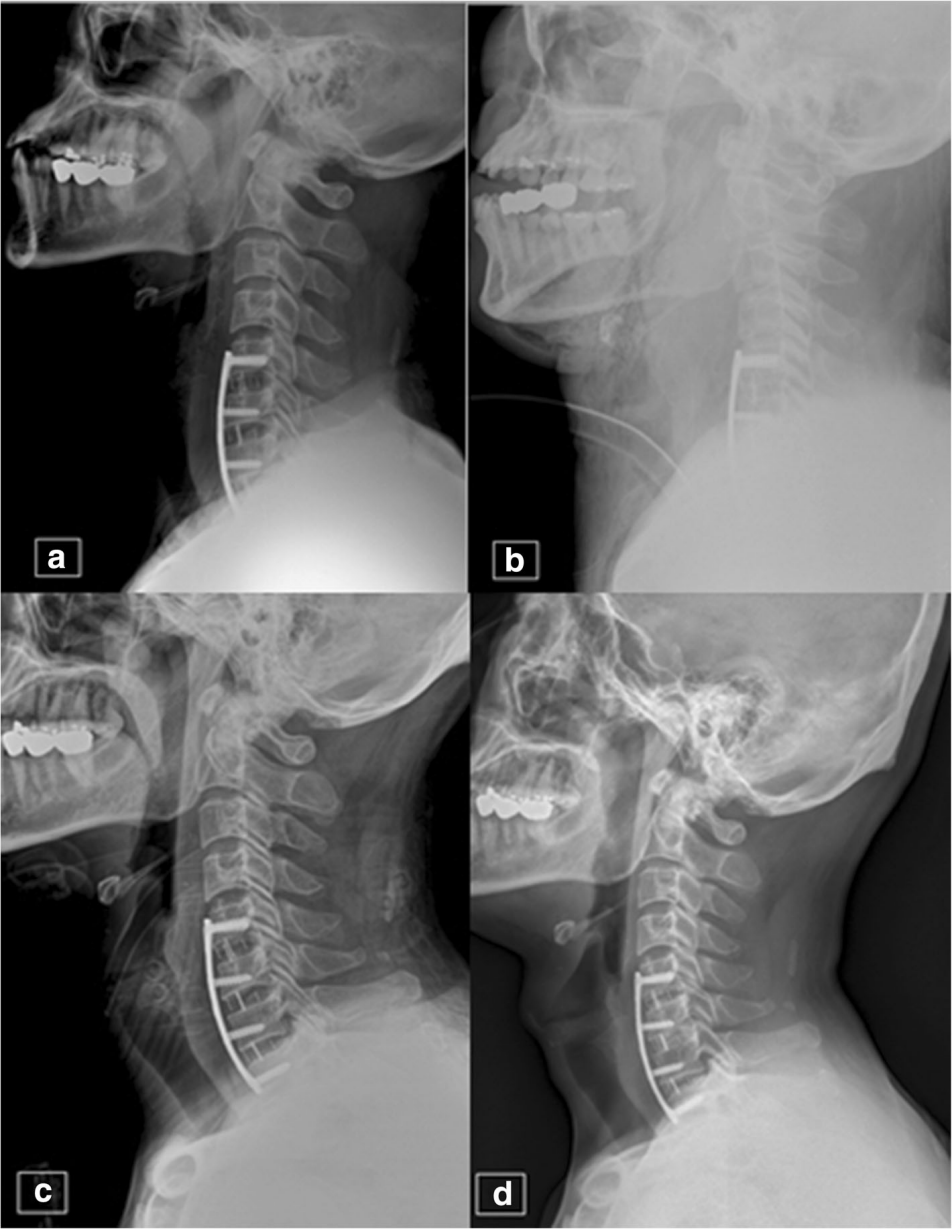
A 47-year-old male patient. **a** Lateral radiograph of the cervical spine on the day of surgery showing anterior cervical discectomy and fusion at C5-6, C6-7, and C7-T1. **b** Lateral radiograph at 10 h postoperatively during adult respiratory failure showing severe prevertebral swelling and cricothyrotomy. The patient had cyanosis and respiration was not responsive. Oxygen saturation was measured at 60–70%. **c** Lateral radiograph at 17 days postoperatively showing decreased prevertebral swelling. The patient was discharged without complications. **d** Lateral radiograph at the 12-month follow-up showing no prevertebral swelling

A 47-year-old man underwent ACF because of herniated intervertebral discs from C5 to T1 and developed acute dyspnea 10 h after surgery. At that time, the patient had a decreased level of consciousness and cyanosis. Blood pressure and O_2_ saturation dropped rapidly to 80/40 mmHg and 60%, respectively. Intubation was attempted twice but failed due to laryngeal edema. We performed a cricothyrotomy while maintaining respiration with an O_2_-reserving bag that allowed recovery of normal breathing. The patient was transferred to the operating room, where the hematoma was removed and the diffuse intramuscular bleeding was stopped under general anesthesia. Four days later, the tube was removed and the patient was able to breathe normally. The patient was discharged from the hospital without any complications 17 days after the ACF surgery (Fig. 2).

## Discussion

Although dyspnea after ACF is not common, it is a severe complication that can develop into hypoxemia and be fatal without appropriate treatment. There are a few reports concerning development of hematoma after anterior cervical surgery. Some authors have considered that vessel damage and intramuscular bleeding due to excessive traction during surgery are the main causes of the hematoma [6–8]. Incorrect hemostasis of the vessels, including the vertebral artery, thyroid artery, and veins of the neck, has been reported as one of the causes of postoperative hematoma in these patients. Further, there are reports of dyspnea being caused by delayed bleeding associated with arterial aneurysms and venous thrombosis [7, 9]. In this study, we could not find the injured vessel during the second surgery, except in one case of injury to the jugular vein. We did not identify any specific risk factor for development of postoperative hematoma, possibly because of our small number of cases. However, we did find that postoperative hematoma could develop without any injury to specific vessels. Therefore, effective hemostasis and avoiding excessive traction intraoperatively are important in all patients. Detection and treatment of postoperative hematoma in the early stages are also important factors that can affect the outcome.

We identified a review article and some case reports on AAO after cervical spine surgery [10–13], but no systematic report on the evaluation and management of AAO in these circumstances. Palumbo et al. classified airway compression into two types. The first is a potentially lethal condition that requires immediate intervention because patients are unable to breathe independently. In such situations, emergent intubation and removal of the hematoma at the bedside are mandatory. The second type of airway compression is not lethal and the patient is able to breathe, although the patient’s breathing should be supported with oxygen while the surgical team attempts intubation and removes the hematoma in the operating room. We perform an emergent airway procedure if the patient is cyanotic, has decreased blood pressure, and decreased oxygen saturation. Patients presenting with a non-lethal airway condition but with anxiety, fear, and increased sweating should be considered for surgery under general anesthesia. However, in the absence of severe respiratory distress, treatment to remove a hematoma could worsen the patient’s condition if breathing is stable and there is no anxiety. Therefore, we ask patients in this state to maintain a sitting position and provide them with oxygen. In addition to close observation, we prepare the surgery team to be on call in case the patient’s condition deteriorates.

Some reports have evaluated dyspnea according to time of occurrence [14–16]. Dyspnea that occurs soon after surgery is thought to be caused by vessel damage, intramuscular bleeding, or tube malfunction. A delayed hematoma occurring 10 days after surgery or even after discharge from hospital may be caused by a pseudoaneurysm or bleeding from the vertebral artery or thyroid artery. A delayed hematoma can cause severe and potentially fatal complications if undetected or not treated. Therefore, it is important to counsel patients regarding this type of complication even though it is uncommon.

An effective and systematic therapeutic approach is required to obtain better results and achieve a better prognosis of following postoperative hematoma-related AAO. Treatment can be selected after early evaluation of the hematoma and the severity of dyspnea. In this study, we attempted to formulate a systematic method of evaluation, an appropriate therapeutic approach, and ways to prevent AAO as a complication of postoperative hematoma.

### Formulation of a therapeutic guideline for AAO after ACF

A patient’s respiratory status can be used to determine the degree of airway compression by thoroughly evaluating the condition of the neck and the patient’s behavior. If the patient shows cyanosis due to inadequate oxygen delivery, emergent intubation should be tried. However, the failure rate of intubation is high because of swelling and adhesion of the vocal cords in the postoperative period. In such situations, emergent cricothyrotomy should be considered instead of reattempting an intubation procedure. If a patient has dyspnea characterized by severe stridor on inspiration and expiration, and an increased breathing rate and elevated blood pressure, he/she should be moved to the operating room immediately with appropriate airway management. Removal of a hematoma in the operating room requires application of adequate hemostatic methods under general anesthesia [6–8].

**Fig. 3:**
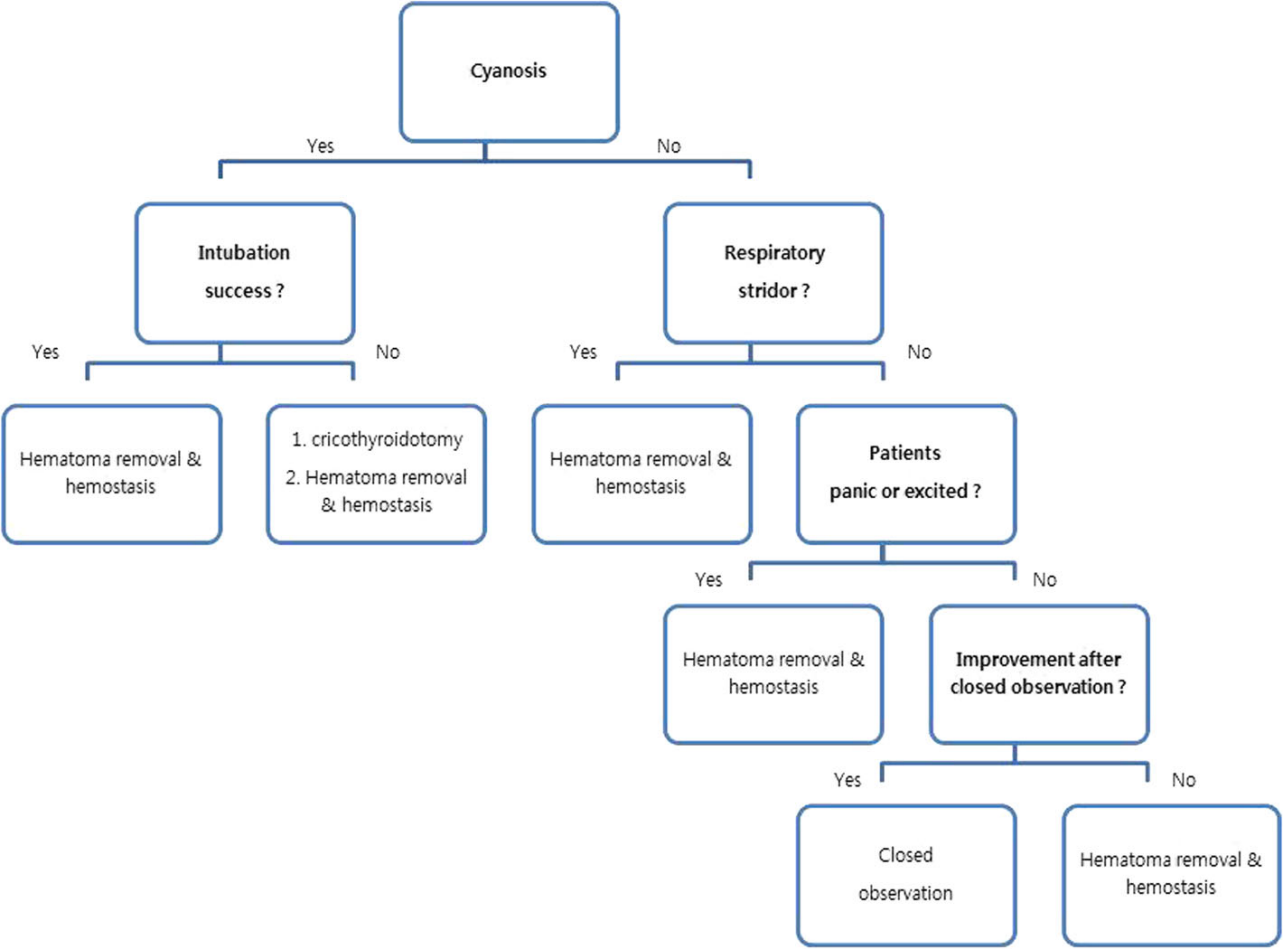
Therapeutic guideline for acute airway obstruction after anterior cervical fusion

The behavior of patients with controlled dyspnea or impaired breathing must be evaluated closely. Specifically, when a patient becomes agitated by fear or anxiety, spontaneous relief of dyspnea is unlikely. Therefore, it is important to take agitated patients to theater immediately, remove the hematoma, find the source of bleeding, and apply a suitable hemostatic method. Patients without fear and anxiety can be stabilized with oxygen and close observation. However, the hematoma should be removed if the patient's condition does not improve [6]. We have developed a guideline for evaluating patients according to their condition and behavior that can be used to guide appropriate treatment (Fig. 3).

Although our study has the limitations of being a retrospective analysis and including only a small number of cases, its results provide the basis for guidelines for initial appropriate treatment of AAO after an anterior cervical spine procedure.

## Conclusions

With rapid recognition and appropriate treatment, there were no long-term complications arising from postoperative hematoma. There were no specific preoperative risk factors for hematoma. To prevent postoperative hemorrhage, performing effective hemostasis during surgery and avoiding excessive traction that can lead soft tissue damage are recommended. Systematic evaluation and appropriate management can be helpful for preventing serious complications after formation of a postoperative hematoma.

## Abbreviations

AAO: Acute airway obstruction
ACF: Anterior cervical fusion
